# Changed epitopes drive the antigenic drift for influenza A (H3N2) viruses

**DOI:** 10.1186/1471-2105-12-S1-S31

**Published:** 2011-02-15

**Authors:** Jhang-Wei Huang, Jinn-Moon Yang

**Affiliations:** 1 Institute of Bioinformatics and Systems Biology, National Chiao Tung University, Hsinchu, 30050, Taiwan; 2 Department of Biological Science and Technology, National Chiao Tung University, Hsinchu, 30050, Taiwan; 3 Core Facility for Structural Bioinformatics, National Chiao Tung University, Hsinchu, Taiwan

## Abstract

**Background:**

In circulating influenza viruses, gradually accumulated mutations on the glycoprotein hemagglutinin (HA), which interacts with infectivity-neutralizing antibodies, lead to the escape of immune system (called antigenic drift). The antibody recognition is highly correlated to the conformation change on the antigenic sites (epitopes), which locate on HA surface. To quantify a changed epitope for escaping from neutralizing antibodies is the basis for the antigenic drift and vaccine development.

**Results:**

We have developed an epitope-based method to identify the antigenic drift of influenza A utilizing the conformation changes on epitopes. A changed epitope, an antigenic site on HA with an accumulated conformation change to escape from neutralizing antibody, can be considered as a "key feature" for representing the antigenic drift. According to hemagglutination inhibition (HI) assays and HA/antibody complex structures, we statistically measured the conformation change of an epitope by considering the number of critical position mutations with high genetic diversity and antigenic scores. Experimental results show that two critical position mutations can induce the conformation change of an epitope to escape from the antibody recognition. Among five epitopes of HA, epitopes A and B, which are near to the receptor binding site, play a key role for neutralizing antibodies. In addition, two changed epitopes often drive the antigenic drift and can explain the selections of 24 WHO vaccine strains.

**Conclusions:**

Our method is able to quantify the changed epitopes on HA for predicting the antigenic variants and providing biological insights to the vaccine updates. We believe that our method is robust and useful for studying influenza virus evolution and vaccine development.

## Background

Influenza viruses occur all over the world and cause significant morbidity and mortality [1]. The surface proteins hemagglutinin (HA) and neuraminidase (NA) are the primary targets of the protective immune system. In circulating influenza viruses, gradually accumulated mutations on the HA, which interacts with infectivity-neutralizing antibodies, lead to the escape of immune system (called antigenic drift). The antibody recognition of HA is highly correlated to the conformation changes on the antigenic sites (epitopes). To quantify a changed epitope escaping from neutralizing antibodies is the basis to study the antigenic drift for the vaccine development [2-5].

Most of methods measuring the antigenic variances on HA focused on amino acid position mutations, such as hamming distance [6] or phylogenic distance [2]. An antibody often utilized complementarily-determining regions (CDRs) to bind two specific sites (called epitopes) on the antigen (HA) [7]. The HA consists of five epitopes and each epitope has ~20 structural neighbour amino acids locating on the protein surface [8]. Recently, few studies discussed the relationships between the epitopes and vaccine efficiency [9].

Here, we have proposed a method to identify the antigenic drift of influenza A by quantifying the conformation change of an epitope. Our method is able to predict antigenic variants of a given pair HA sequences which are often a vaccine strain and a circulating strain. Our model was evaluated to measure the antigenic drifts and vaccine updates on 3,331 circulating strains (from year 1982 to 2009) and to predict the antigenic variants on two data sets (i.e. 343 and 31,878 HI assays). These results demonstrate that our model is able to reflect the biological meanings and can explain the selections of WHO vaccine strains.

## Materials and methods

**Figure 1:**
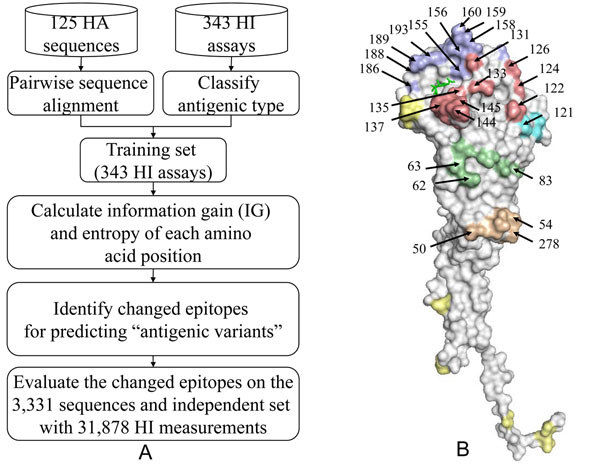
**Overview of our method for the antigenic drift**. (A) The overview of our method. (B) The structural locations of selected 64 critical amino acid positions on five epitopes (Epitope A in red; B in purple; C in orange; D in cyan; E in green). The sialic acid is in green. All structures are presented by using PyMOL.

Figure 1 presents the overview of our method for the antigenic drift of human influenza A (H3N2) viruses by quantifying changed epitopes. We first identified the critical amino acid positions based on both the antigenic variant and genetic diversity. We then measured a changed epitopes by calculating the accumulated conformation change based on amino acid mutations on an epitope. Finally, we evaluated our model for predicting antigenic variants and selecting the WHO vaccines.

### Changed epitopes

The changed epitope is the core of our method. Here, we defined a changed epitope as follows: an antigenic site (epitope) on HA with accumulated amino acid mutations induces the conformation change to escape from the neutralizing antibody. The conformation change of a mutation depends on its position on HA structure and the mutation rate during 40 years. A changed epitope can be considered as a "key feature" for measuring antigenic variants of a pair HA sequences. Here, a changed epitope can be used to predict antigenic variants and antigenic drifts for the selections of vaccine strains. The definition of five epitopes including 131 positions was proposed by Wilson *et al.*[8] and Bush *et al.*[2].

### Data sets

To describe and evaluate the ability of the changed epitopes for predicting antigenic variants, we collected hemagglutination inhibition (HI) assays, describing the antigenic variants and similar viruses of the current global influenza surveillance system. The HI assay describes whether one (e.g. circulating) strain will be recognized by an antibody against the vaccine strain. We collected 343 H3N2 virus HI assays (Data available at http://gemdock.life.nctu.edu.tw/influenza/File1.xls) with 125 HA sequences from Weekly Epidemiological Record (WER) [10], World Health Organization (WHO) collaborating center [11] and related publications [12-14] (Table S1 in additional file 1). Each pair includes the HI assay value (i.e. antigenic distance) and a pair of HA sequences (329 amino acids). In general, an influenza vaccine should be updated if an antigenic distance is more than 4.0 between the current vaccine strain and the circulating strain in next season [3][15]. Among 343 pairs of HA sequences, 225 pairs with antigenic distance ≥ 4 are considered as "antigenic variants" and 118 pairs are considered as "similar viruses". For example, the antigenic distance of the pair HA sequences, A/England/42/72 and A/PortChalmers/1/73, is 12 and this pair is considered as "antigenic variants". Conversely, the antigenic distance of the pair HA sequences, A/Wuhan/359/95 and A/Nanchang/933/95, is 1 and this pair is considered as "similar viruses". In addition, we prepared another HI assay data set to independently evaluate our model for predicting antigenic variants proposed by Smith *et al.*[*3*]. We assume that a virus-pair in the same antigenic group is considered as a "similar viruses" pair and a virus-pair in different groups is considered as "antigenic variants" pair. Finally, we yielded 31,878 HI measurements from the supporting materials [3].

To study the antigenic drifts and WHO vaccine updates, we collected 3,331 HA sequences (Data available at http://gemdock.life.nctu.edu.tw/influenza/File2.xls) from influenza virus resource [16] and influenza sequence database [17]. These sequences were assigned into 38 influenza seasons according to their collection dates.

### Identify antigenic critical positions on HA

Recently, we proposed a method to identify antigenic critical positions [5] by utilizing both antigenic variants and genetic diversity. The Shannon entropy and information gain (IG) were used to measure genetic diversity and antigenic discriminating score for amino acid positions on HA, respectively. Here, we based on these rules to select 64 amino acid positions as the critical positions (Table S2 in additional file 1).

### Models for antigenic variants based on changed epitopes

To address the issue of measuring accumulated mutations on an epitope to escape from neutralizing antibody, we proposed 4 models considering the number of amino acid mutations on 329 amino acids and 64 selected critical positions of HA (Table 1). Models one and two regarded an epitope as "changed" if there are more than 1 and 2 mutations within an epitope, respectively, based on 329 amino acids. A changed epitope of Model three is defined as two amino acid mutations on 64 critical positions. Models one, two, and three regarded a pair HA sequences as "antigenic variants" if there are more than two changed epitopes. Conversely, one changed epitope is viewed as "similar viruses".

**Table 1:** Summary of four models

Model	Regarding HA positions	Changed epitope	Antigenic variants
Model one	329 positions	≥1 mutation	≥2 changed epitopes
Model two	329 positions	≥2 mutations	≥2 changed epitopes
Model three	64 selected positions	≥2 mutations	≥2 changed epitopes
Model four	64 selected positions	≥3 mutations (epitope B) ≥2 mutations (others)	≥1 (epitopes A or B) ≥2 (others)

Model four treated one changed epitope (A or B) as "antigenic variants". Epitopes A and B, which are near to the receptor binding site, often play the key role for escaping from neutralizing antibody. Here, the epitopes A and B (denoted as "B+") were regarded as "changed" if there are more than 2 and 3 mutations, respectively. For the pair A/Mississippi/1/85 and A/Leningrad/360/86 (Table 2), the numbers of mutations were 1, 3, 0, 1, and 1 on epitopes A, B, C, D and E, respectively. The numbers of changed epitopes for Models one and two are 4 (epitopes A, B, D, and E) and 1 (epitope B), respectively. Models three and four regarded the epitope B as a changed epitope because these three mutations (i.e. positions 156, 159 and 188) were the selected critical positions.

**Table 2:** The changed epitopes and mutations of 11 virus-pairs under 4 models

Virus A	Virus B	Type^1^	Changed epitopes				HD^2^	Mutation positions				
	
			Model one	Model two	Model three	Model four		Epitope A	Epitope B	Epitope C	Epitope D	Epitope E
A/PortChalmers/1/73	A/Singapore/4/75	S	ABCDE	B	B	B	9	**126**^3^	**160, 189**	**278**	**242**	**83**
A/Nanchang/933/95	A/NewYork/43/96	S	ABCE	E	none	none	6	**122**	190	**275**		57, 92, **262**
A/Alaska/10/95	A/France/75/97	S	ABCDE	BC	none	none	12	**135**	**128**, 165	**275,** 312	226	**262**
A/Sydney/5/97	A/Ireland/10586/99	S	ABDE	ABD	none	none	7	**137**, 142	192, 194		**172**,226	57
A/Mississippi/1/85	A/Leningrad/360/86	V	ABDE	B	B	B+	6	138	**156, 159, 188**		226	88
A/Guizhou/54/89	A/Beijing/353/89	V	ABC	A	A	A	5	**135, 144, 145**	**159**	44,		
A/Wellington/1/2004	A/Victoria/505/2004	S	ABDE	AD	none	none	10	138, **145**	**189**		219, 226, 227	94
A/Shangdong/9/93	A/Pennsylvania/9/93	S	ABCD	CD	C	C	12	**135**	**164**	**53, 276**	214, 219, 226, 229, 238	
A/England/42/72	A/PortChalmers/1/73	V	BDE	B	B	B+	6		**160, 188, 193**		208	**63**
A/NewYork/55/2004	A/Anhui/1239/2005	V	ABD	B	B	B+	7	138,	**156, 160, 193**		219,	138,
A/Shanghai/16/89	A/Beijing/353/89	V	AB	A	A	A	3	**135, 145**	**159**			

^1^V represents antigenic variant and S represents similar virus.

^2^ denote hamming distance of a pair sequences

^3^ Bold is the antigenic critical position.

Finally, we compared our models with two related methods [4,8] for predicting antigenic variants. Wilson & Cox [8] suggested that a viral variant usually contains more than 4 residue mutations located on at least two of the five epitopes. Lee & Chen [4] proposed a model based on the hamming distance (HD) of 131 positions on five epitopes to predict antigenic variants. Their models predicted a pair of HA sequences as "antigenic variants" if the number of mutation is more than 6.

### Variant ratio for measuring the antigenic drift

We used the variant ratio (VR) to measure the vaccine efficiency on year *y*. The VR is defined as , where *N_y_* is total number of circulating strains in the year *y* and *V_y_* is the number of circulating strains which are "antigenic variants" against the vaccine strain in the year. Here, we considered an influenza vaccine should be updated and the antigenic variants are emerging if the VR value is ≥ 0.5.

## Results

### Antigenic critical positions

In this study, we followed our previous work to select the critical positions [5] having high IGs, statistically derived from 343 HI assays, and high entropies, which were calculated using 125 HA sequences. 64 positions on HA were selected as critical positions (Table S2 in additional file 1). Among these 64 critical positions, 54 positions locate on the epitopes (54/64) and 53 positions locate on the HA surface (Fig. 1B). Additionally, 13 and 42 of these 64 critical positions were the positive selections [2] and cluster substitutions [3], respectively.

### Changed epitopes for antigenic variants

**Figure 2:**
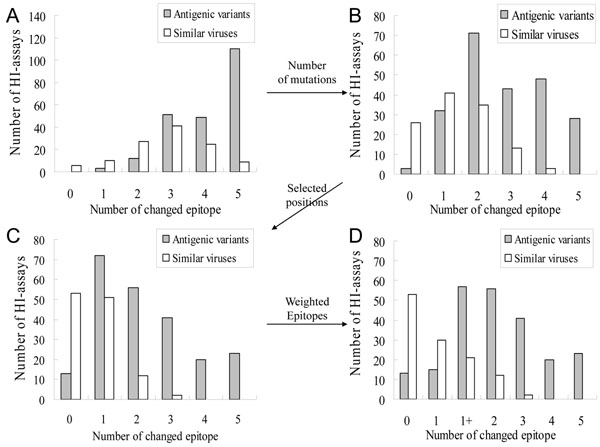
**The relationships between number of changed epitopes and antigenic variants on 4 models.** (A) The first model considered an epitope as changed if there is at least one mutation within it. (B) The second model considered an epitope as changed if there are at least two mutations within it. (C) The third model considered an epitope as changed if there are at least two critical mutations within it. (D) The fourth model was derived from model three and further defined "1+" type if there are at least 2 and 3 critical mutations in epitope A and B. respectively.

**Figure 3:**
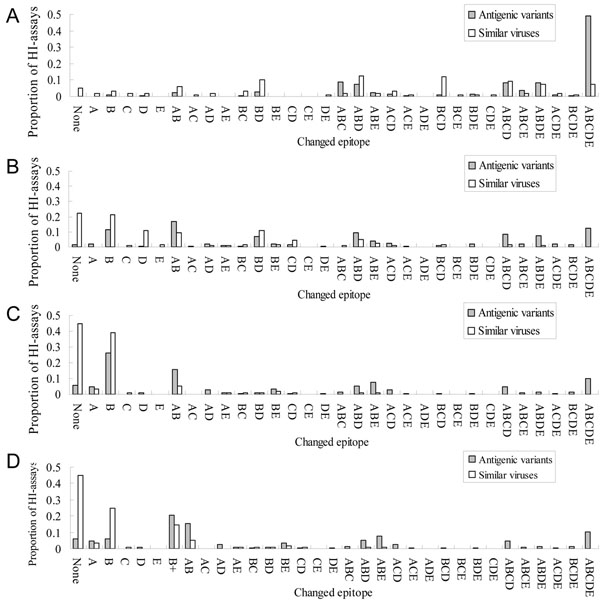
**The changed-epitope composition and antigenic variants on 4 models.** (A) Model one. (B) Model two (C) Model three. (D) Model four.

Currently, several methods measured a changed epitope to escape from neutralizing antibody [8]. Here, we utilized the degree of accumulated mutations within an epitope to evaluate a changed epitope according to 329 positions and 64 selected positions. Figures 2 and 3 show the relationships between changed epitopes and antigenic variants on 4 models.

### Models one and two: Changed epitopes on 329 positions

Figures 2A (Model one) and 2B (Model two) show the relationships between number of changed epitopes and "antigenic variants" on 343 pair HA sequences with HI assays. Among these 343 pairs for Model one, the changed epitopes of 225 "antigenic variants" pairs range from 1 to 5 and the changed epitopes of 118 "similar viruses" pairs range from 0 to 5. Among 34 similar viruses with more than 4 changed epitopes for Model one, we observed the following results: (1) the average number of changed epitopes was 4.2; (2) the average number of changed epitopes with only one mutation was 2.02 and 33 pairs have more than one changed epitope with only one mutation. For example, the virus pair, A/PortChalmers/1/73 and A/Singapore/4/75, has four changed epitopes with one mutation (i.e. Epitopes A, C, D, and E) (Table 2). In general, these 34 similar viruses should be regarded as "antigenic variants" because there are more than four changed epitopes. This result shows that the Model one is not reasonable.

For Model two, the average number of changed epitopes was 2.2 for these 34 similar viruses.  According to the distribution (Figure 2B), Model two achieved the highest accuract if more that two changed epitopes was considered as "antigenic variants".  The accuracies were 74.9% (257/343) and 92.2% (29410/31878) for predicting antigenic variants on the training set and independent set, respectively.  This result was similar to the previous work [8].

### Model three: Changed epitopes on 64 selected positions

Model three considered a changed epitope when the number of mutations on the 64 selected critical positions is more than 2. In Model two, the numbers of "antigenic variants" and "similar viruses" with ≥ 3 changed epitopes were 119 and 16, respectively (Fig. 2B). The averages of changed epitopes with ≥ 2 mutations on 329 positions for "antigenic variants" and "similar viruses" were 3.8 and 3.2, respectively. The averages of changed epitopes with ≥ 2 mutations on 64 selected critical positions for "antigenic variants" and "similar viruses" were 3.2 and 1.5, respectively (Fig. 2C). These results show that Model three using mutations on 64 critical positions is better than Model two to discriminate "antigenic variants" from "similar viruses". For the "similar viruses", A/Alaska/10/95 and A/France/75/97, there are 12 mutations to drive zero changed epitope because no epitope with ≥ 2 mutations on selected 64 positions (Table 2).

Three HA/antibody complex structures can be used to provide structural evidences for the changed epitopes [18] (Fig. S1 in additional file 1). Among these complexes, two antibodies bind to epitopes A and B (PDB code 1KEN [19] and 2VIR [20]), while the third binds to epitopes C and E (PDB code 1QFU [21]). The antibodies consistently bind to two epitopes and this result agrees to Models two and three. HA/antibody structures and Models two and three show that two position mutations often induce the conformational change of an epitope to escape from the antibody recognition. However, the numbers of changed epitopes of 48 "similar viruses" pairs are 2 (35 pairs) and 3 (13 pair) for Model two (Fig. 2B). Conversely, 14 "similar viruses" pairs have more than 2 changed epitopes for Model three (Fig. 2C).

### Model four

Among 72 "antigenic variants" pairs with one changed epitope based on Model three, 70 pairs change on epitopes A or B. The single changed epitope on A or B, which can cause "antigenic variants", agreed to HA/antibody complex structures and the experiments. The receptor binding site, surrounded by epitopes A and B, is a basis for HA protein for the neutralizing mechanism [19,22] (Fig. 1B).

Based on this observation, the epitopes A and B play a key role for neutralizing antibodies. Model four based on Model three considered a pair HA sequences as "antigenic variants" when ≥ 2 changed epitopes or ≥ 1 changed epitope on A or B. In Model four, a pair HA sequences with ≥ 3 mutations on 64 critical positions for the epitope B is regarded as "antigenic variants". Thus, we annotated a virus-pairs with single changed epitope on A or B as "1+" type (Fig. 3D). For example, the pair, A/Guizhou/54/89 and A/Beijing/353/89, occurs the changed epitope on A (i.e. mutation positions 135, 144 and 145) (Table 2). The accuracies of Model four were 81.6% and 94.0% on the training set and independent set, respectively. This model outperformed two compared methods, i.e. Wilson & Cox (89.7%) [8] and Lee & Chen (92.4%) [4], on the independent data set (Fig. S2 in additional file 1).

**Figure 4:**
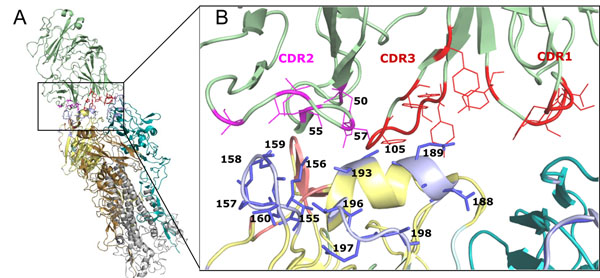
**The HA/antibody structure and interface.** (A) The antibody (pale green) and HA trimer (PDB code 1KEN). (B) The interface of the antibody and HA. The selected critical positions on epitope B and the CDRs in the heavy (red) and light (pink) chains of the antibody are labelled.

In the HA/antibody structure complex (PDB code 1KEN [19]), the antibody binds on epitopes A and B using two CDRs (i.e. CDR1 and CDR3) on the heavy chain and one CDR (i.e. CDR2) on the light chain (Fig. 4). The interface of antibody and HA consists of 13 and 5 contacted residues locating on epitopes B and A, respectively. Among these 13 positions, 7 positions were selected as critical positions. Based on Model four, 46 "antigenic variants" pairs have one changed epitope B with 3 mutations on epitope B, denoted as "B+". This result suggested a single changed epitope B can cause antigenic variants. For example, the pair virus strains, A/NewYork/55/2004 and A/Anhui/1239/2005, have three critical mutations on epitope B (i.e. positions 156, 160 and 193) (Table 2). According to the HA/antibody structure (Fig. 4), the residue 156 interacts to CDR2 (position 55 on the antibody) and the residue 193 interacts with three residues on CDR2 (positions 50, 55 and 57) and one residue on CDR3 (position 105). This structure suggested that mutations on residues 156, 160 and 193 can induce the conformation change on epitope B to escape from CDR2 and CDR3 of the neutralizing antibody.

### Antigenic drift and epitope evolution

We utilized the changed epitopes to study the antigenic drift on 3,331 circulating strains ranging from 1982 to 2009 (38 influenza seasons). One of WHO surveillance network's purposes is to detect the emergence and spread of antigenic variants that may signal a need to update the composition of influenza vaccine [1,3]. Here, we considered an emerging antigenic variant according to WER strain, which was the dominant strain in each influenza season [6] (Table S1 in additional file 1). For a selected season, we applied Model four, measuring changed epitopes for the pairs between the vaccine and circulating strains for "antigenic variants", and the variant ratio (VR) to detect the emerging antigenic variants.

**Figure 5:**
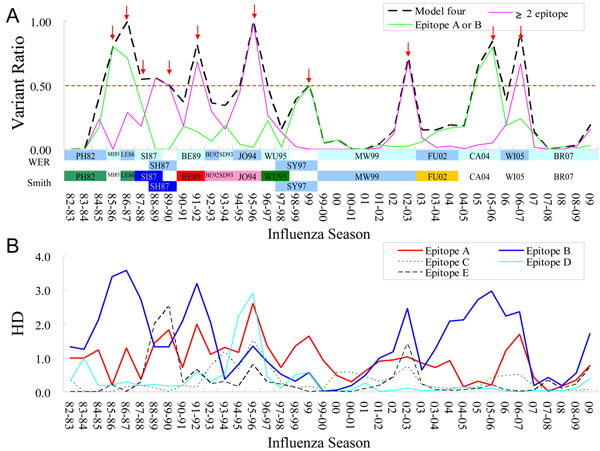
**The epitope evolution and antigenic drift.** (A) The distributions of variant ratios of WER and Smith vaccine strains from 1982-1983 to 2009 seasons. 10 seasons with emerging variants and followed by the update of WER strain in the next season are labelled (red arrow) (B) The average hamming distances (HD) of 5 epitopes from 1982-1983 to 2009 seasons.

Among 38 seasons (1982~2009), our model detected 12 seasons with emerging antigenic variants (VR ≥ 0.5) and 10 of them followed by the update of WER strain in the next season (Fig. 5A). For example, the 85-86 season, 80% of the circulating strains with changed epitope "B+" (Fig. 5B), is the first emerging antigenic variants and the WER strain updated in the next season (i.e. from A/Mississippi/1/85 to A/Leningrad/360/86). In addition, among seven "emerging antigenic variants" seasons (matching WHO vaccine updates), four seasons (i.e. 89-90, 91-92, 95-96 and 02-03) matched the antigenic cluster transitions proposed by Smith *et al*. [3]. The other three seasons, which were detected by one changed epitope on A or B, are consistent to the WER strain updates (i.e. 87-88, 85-86 and 99). These results suggested that "emerging antigenic variants" with ≥ 2 changed epitopes may cause the major antigenic drift while "emerging antigenic variants" with one changed epitope on A or B may cause the minor antigenic drift.

To observe the epitope evolution, Figure 5B illustrates the hamming distance (HD) on 64 critical positions of five epitopes. For example, the VR of the season 85-86 was 0.8 (Fig. 5A) and the epitope with the largest HD was epitope B (HD is 3.4). For 16 seasons with WER strain updates, the average HDs of epitopes A, B, C, D and E were 1.2, 2.1, 0.4, 0.4 and 0.5 respectively. These results showed that epitopes A and B change more frequently in vaccine update seasons and they play a key role for antigenic drift.

## Discussion

According to the distribution of antigenic variants of Model four (Fig. 3D), it is interesting that the major pairs (209/225 pairs) of the antigenic variants have the changed epitope on epitopes A or B which are closed to the receptor binding site. Furthermore, many experiments suggested that the occlusion of the receptor binding site by antibodies bound to the HA molecule forms the dominant neutralizing mechanism [19,22]. These results implied that a pair of viruses often is "similar virus" if the epitopes A and B are not changed.

Among 225 "antigenic variants" pairs, 13 pairs have no changed epitopes (Table S3 in additional file 1). 11 pairs of these 13 pairs have contradict antigenic types by two antiseras, which suggested a more powerful experimental assay is required to verify the antigenic types. For example, the antibody against the A/Alaska/10/95 strain can't inhibit the A/Idaho/4/95 strain; while the antibody against the A/Idaho/4/95 strain inhibits the A/Alaska/10/95 strain.

## Conclusions

This study demonstrates our model is robust and feasible for quantifying the changed epitopes. According to the distribution of antigenic variants in HI assays and HA/antibody complex structures, we found that two critical position mutations with high genetic diversity and antigenic scores can induce the conformation change of an epitope. Epitopes A and B, closing the receptor binding site of HA, play a key role for neutralizing antibodies. In addition, two changed epitopes often drive the antigenic drift and can be used to explain the selections of 24 WHO vaccine strains. We believe that our method is useful for the vaccine development and studying the evolution of human influenza A virus.

## Competing interests

The authors declare that they have no competing interests.

## Authors' contributions

Conceived and designed the experiments: JWH and JMY. Performed the experiments and analyzed the data: JWH and JMY. Wrote the paper: JWH and JMY

## Supplementary Material

Additional file 1 The supplementary information Click here for file
