# Supplementary material for: Changed epitopes drive the antigenic drift for influenza A (H3N2) viruses
Source: BMC Bioinformatics. 2011 Feb 15;12(Suppl 1):S31. doi: 10.1186/1471-2105-12-S1-S31 (PMC3044287; doi:10.1186/1471-2105-12-S1-S31)
Supplement: Additional file 1 — The supplementary information [file 1471-2105-12-S1-S31-S1.pdf]

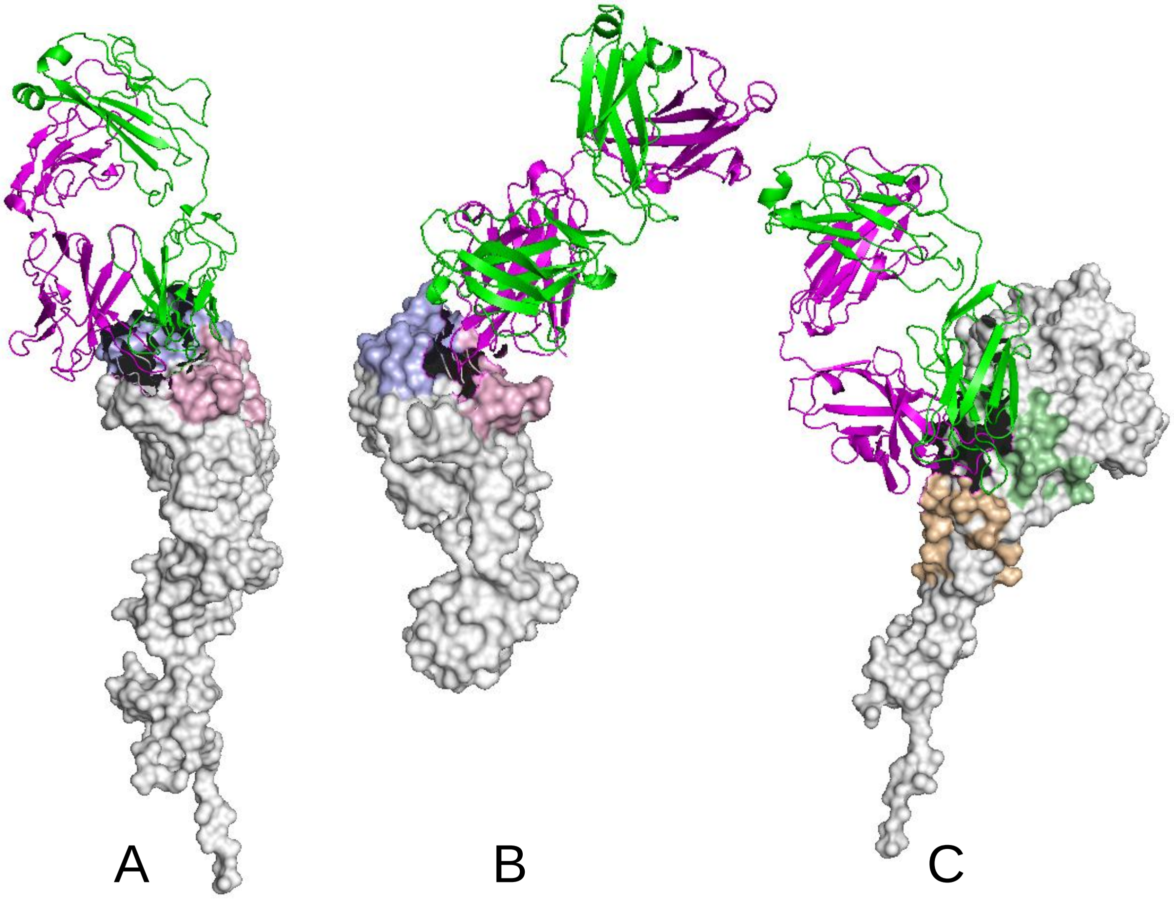

Supplementary Figure S1 : The three HA-antibody complex structures. PDB codes are (A) 1KEN [1], (B) 2VIR [2], and (C) 1QFU [3].

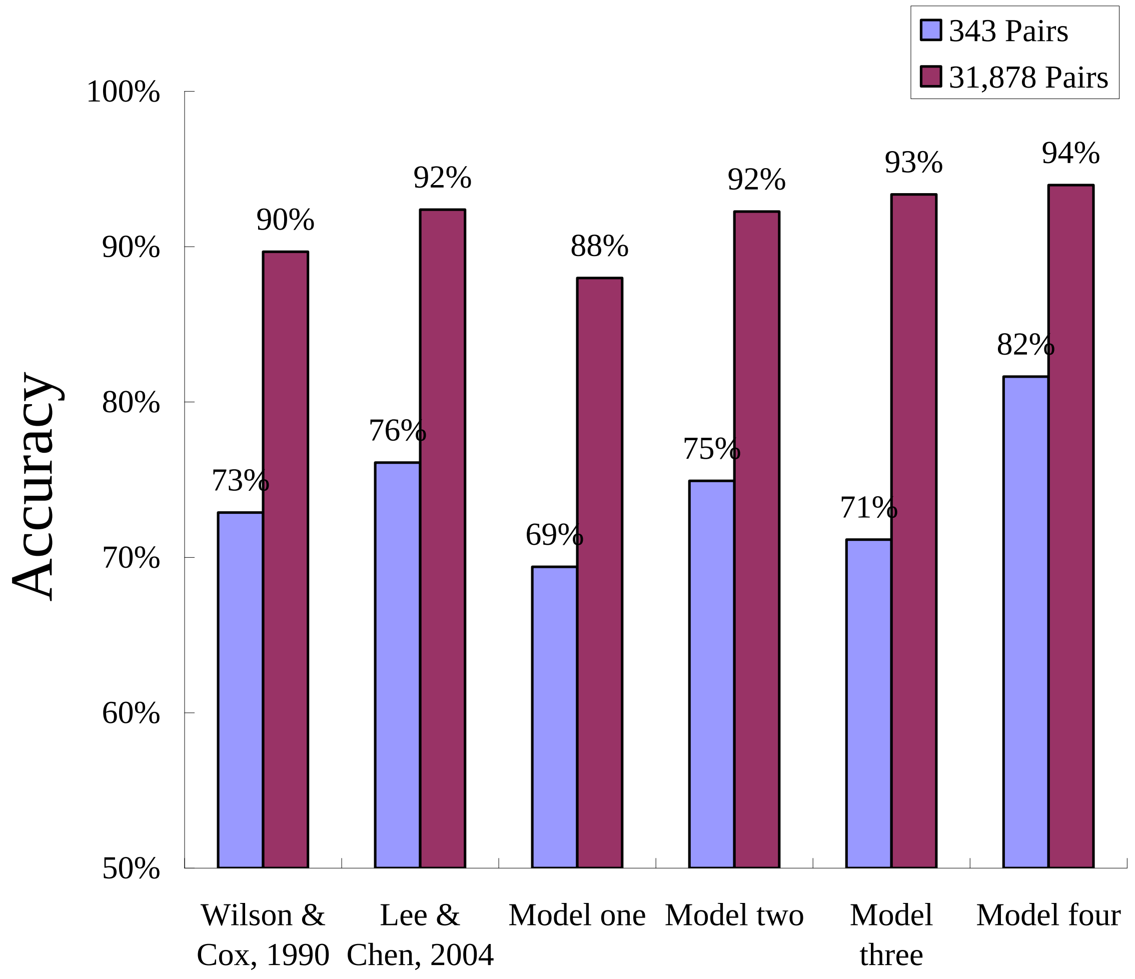

Supplementary Figure S2 : Compare our method with other two methods on predicting antigenic variants on two data sets.

Supplementary Table S1 : The number of HI assays and number of sequences in Smith's dataset and WER strains from 1968 to 2009

Year	HI-assay data set	Smith's data set [4]	WER strain[1]
1968	0	4	A/Hong Kong/1/68
1969	0	3	A/Hong Kong/1/68
1970	1 [5]	2	A/Hong Kong/1/68
1971	2 [5]	4	A/Hong Kong/1/68
1972	2 [5]	5	A/Hong Kong/1/68
1973	2 [5]	4	A/England/42/72
1974	3 [5]	5	A/Port Chalmers/1/73
1975	6 [5]	3	A/Port Chalmers/1/73
1976	7 [5]	6	A/Victoria/3/75
1977	4 [5]	5	A/Victoria/3/75
1978	0	0	A/Texas/1/77
1979	0	0	A/Texas/1/77
1980	6 [5]	2	A/Bangkok/1/79
1981	0	1	A/Bangkok/1/79
1982	3 [5]	4	A/Bangkok/1/79
1983	5 [5], 23[6]	1	A/Phillipines/2/82
1984	0	1	A/Phillipines/2/82
1985	3 [5]	4	A/Phillipines/2/82
1986	9 [5]	2	A/Christchruch/4/85, A/Mississippi/1/85
1987	3 [5]	3	A/Leningrad/360/86
1988	17 [5]	4	A/Sichuan/2/87
1989	0	16	Si/87; Sh/87
1990	10 [5]	5	A/Shanghai/11/87
1991	5 [5]	17	A/Beijing/353/89
1992	5 [5]	45	A/Beijing/353/89
1993	21 [5]	43	A/Beijing/32/92
1994	6 [5]	10	A/Shangdong/9/93
1995	3 [5], 20 [7]	15	A/Johannesburg/33/94
1996	12 [5]	10	A/Johannesburg/33/94
1997	28 [8]	9	A/Wuhan/359/95
1998	3 [5]	4	A/Wuhan/359/95; A/Sydney/5/97
1999	2 [5]	3	A/Sydney/5/97
2000	0	1	A/Moscow/10/99 [2]
2001	11 [9]	3	A/Moscow/10/99
2002	0	3	A/Moscow/10/99
2003	11[5], 20 [10]	6	A/Moscow/10/99
2004	4 [5], 17 [10]	0	A/Fujian/411/2002
2005	14 [5],1 [10]	0	A/Calfornia/7/2004
2006	11 [5], 9 [10]	0	A/Calfornia/7/2004; A/Wisconsin/67/2005
2007	3 [5], 31 [11]	0	A/Wisconsin/67/2005; A/Brisbane/10/2007
2008	0	0	A/Brisbane/10/2007
2009	0	0	A/Brisbane/10/2007
Total	343 pairs	253 sequences	

[1] WER strain is the dominant virus in influenza season reported by WHO.

[2] The widely used vaccine strain A/Panama/2007/99 was used instead in following years.

Supplementary Table S2 : The list of 64 selected critical positions

	List of critical positions
Epitope A	122,124,126,131,133,135,137,140,143,144,145,146
Epitope B	128,155,156,157,158,159,160,164,186,188,189,193,196,197,198
Epitope C	50,53,54,275,276,278,307
Epitope D	121,172,174,201,207,213,216,217,230,242,244,248
Epitope E	62,63,75,78,82,83,260,262
Other area	2,3,9,25,31,199,202,222,225,326

Supplementary Table S3 : The 13 antigenic variants without changed epitopes

Virus A	Virus B	Type (A to B)[1]	Type (B to A)[2]	HD	Epitope A	Epitope B	Epitope C	Epitope D	Epitope E
A/Alaska/10/95	A/Idaho/4/95	V	S	3	145	165	312		
A/Alaska/10/95	A/Wuhan/359/95	V	S	5	135	165, 194	275		262
A/Alaska/10/95	A/Hongkong/55/95	V	S	7	135, 138	165	275	167, 226	262
A/Alaska/10/95	A/Shanghai/9/95	V	S	7	135	165, 193, 194	275	226	262
A/Anhui/1239/2005	A/Wisconsin/67/2005	V	S	5	122, 138	160			
A/Fujian/140/2000	A/Chile/6416/2001	V	S	12	144	186, 194	273	226, 246, 247	
A/Hong_Kong/1/94	A/Guangdong/25/93	V	S	8	124		47, 299	96, 216, 219, 226	92
A/Panama/2007/99	A/Chile/6416/2001	V	S	7	144	186		246	
A/Wellington/1/2004	A/Singapore/68/2004	V	S	9	145	189	50	226, 227	94
A/Wellington/1/2004	A/Victoria/513/2004	V	S	5	145	186, 190		167, 226	
A/Wellington/1/2004	A/Wisconsin/19/2004	V	S	5	138, 145	186	278	226	
A/Fujian/140/2000	A/NewYork/55/2001	V	V	12	144	186, 194	273	226, 229, 247	
A/Victoria/3/75	A/Victoria/112/76	V	V	2				229	

[1] the antigenic type of virus B relative to antisera against virus A.
[2] the antigenic type of virus A relative to antisera against virus B.

References

1. Barbey-Martin C, Gigant B, Bizebard T, Calder LJ, Wharton SA, Skehel JJ, Knossow M: **An antibody that prevents the hemagglutinin low pH fusogenic transition**. *Virology* 2002, **294**(1):70-74.
2. Bizebard T, Gigant B, Rigolet P, Rasmussen B, Diat O, Bosecke P, Wharton SA, Skehel JJ, Knossow M: **Structure of influenza virus haemagglutinin complexed with a neutralizing antibody**. *Nature* 1995, **376**(6535):92-94.
3. Fleury D, Barrere B, Bizebard T, Daniels RS, Skehel JJ, Knossow M: **A complex of influenza hemagglutinin with a neutralizing antibody that binds outside the virus receptor binding site**. *Nature Structural Biology* 1999, **6**(6):530-534.
4. Smith DJ, Lapedes AS, de Jong JC, Bestebroer TM, Rimmelzwaan GF, Osterhaus AD, Fouchier RA: **Mapping the antigenic and genetic evolution of influenza virus**. *SCIENCE* 2004, **305**(5682):371-376.
5. WHO: *Weekly Epidemiological Record* 1970-2007, **45, 46, 48, 49, 50, 51, 52, 55, 57, 58, 60, 61, 62, 63, 65, 66, 67, 68, 69, 70, 71, 73, 74, 78, 79, 80, 71, 82**.

6. Both GW, Sleigh MJ, Cox NJ, Kendal AP: **Antigenic Drift in Influenza Virus-H3 Hemagglutinin from 1968 to 1980 - Multiple Evolutionary Pathways and Sequential Amino-Acid Changes at Key Antigenic Sites**. *J Virol* 1983, **48**(1):52-60.
7. Ellis JS, Chakraverty P, Clewley JP: **Genetic and antigenic variation in the haemagglutinin of recently circulating human influenza A (H3N2) viruses in the United Kingdom**. *Arch Virol* 1995, **140**(11):1889-1904.
8. Centers for Disease Control and Prevention: **Information for FDA vaccine advisory panel meeting. Atlanta: The Centers**. 1997:p. 30.
9. Coiras MT, Aguilar JC, Galiano M, Carlos S, Gregory V, Lin YP, Hay A, Perez-Brena P: **Rapid molecular analysis of the haemagglutinin gene of human influenza A H3N2 viruses isolated in Spain from 1996 to 2000**. *Archives of virology* 2001, **146**(11):2133-2147.
10. Centers for Disease Control and Prevention: **Information for the Vaccines and Related Biological Products Advisory Committee, CBER, FDA**. 2003-2007:p. 28, p.18, p.19, p.17.
11. Centers for Disease Control and Prevention: **Options for Live Attenuated Influenza Vaccines (LAIV) in the Control of Epidemic and Pandemic Influenza, Geneva, 12-13 June, 2007**. 2007:p. 11.
